# Lead poisoning with encephalic and neuropathic involvement in a child: case report

**DOI:** 10.11604/pamj.2022.42.276.33007

**Published:** 2022-08-12

**Authors:** Latifa Rouzi, Hecham Elhamri, Samira Kalouch, Siham Salam, Bouchra El Moutawakil, Hanan Chaoui, Narjis Badrane, Mohammed Fekhaoui, Zineb Jouhadi

**Affiliations:** 1 Department of Zoology and Animal Ecology, Scientific Institute, Mohammed V University in Rabat, Rabat, Morocco,; 2 Department of Toxicology and Hydrology, National Institute of Hygiene, Rabat, Morocco,; 3 Department of Pediatric Anesthesiology and Intensive Care Unit, Children's University Hospital Ibn Rochd, University Hassan 2, Casablanca, Morocco,; 4 Department of Pediatric Radiology, Children's University Hospital Ibn Rochd, Casablanca, Morocco,; 5 Department of Neurology, Hospital Ibn Rochd, Hassan 2 University, Casablanca, Morocco,; 6 Poison Control Centre of Rabat, Rabat, Morocco,; 7 Department of Pediatric Infectious Diseases, Children's University Hospital Ibn Rochd, Hassan 2 University, Casablanca, Morocco

**Keywords:** Lead poisoning, children, encephalopathy, neuropathy, case report

## Abstract

Lead is a toxic substance in our environment that affects adults and children of all socioeconomic backgrounds, lead poisoning is one of the most common exposures that can cause inter alia significant neurological and functional damage in humans. Children are particularly vulnerable because of the effects of the toxicity on their developing nervous systems with potentially irreversible consequences. We report a case of severe lead poisoning encephalo-neuropathy in a 3-year-old girl, admitted for progressive paraplegia, swallowing disorders, and aphasia. A multitude of investigations undertaken could not explain her atypic symptoms, so anamnesis was redone in the sense of a toxic origin, we found a notion of pica, and a traditional herbalist father, so probably consumption of medications based on traditional medicine products. A venous blood lead level (BLL) was extremely elevated at 176.4 μg/l. The child was treated with an oral chelator succimer (SUCCICAPTAL). During the two following months in the intensive care unit, the child showed progressive respiratory distress and worsening signs of the nervous system. Despite treatment and the use of lead chelators, the patient died due to septic shock. Lead is highly toxic even at very low exposure levels, at high levels of exposure, it can damage the reproductive organs, immune system, liver and kidneys. in children, it can affect neurocognitive and behavioral development that could be irreversible. Peripheral and central nervous system damage should be considered as a possible manifestation of lead poisoning.

## Introduction

Throughout human history, lead has been both a necessity and an evil. It has been used widely in various products due to its physicochemical properties, abundance, and low cost. Lead can enter the human body via air inhalation and food ingestion. direct ingestion of contaminated soils and lead paint by hand-to-mouth activity (pica) is a common way of lead exposure in humans; especially in children [1]. Exposure to lead increases blood lead levels that damages many organ systems, cardiovascular, skeletal, reproductive organs, kidneys, and liver, but the nervous system is the most vulnerable, especially in young children. Patients with high blood lead levels may experience severe, intractable abdominal colic pain, anemia, weakness, paralysis, high blood pressure, and renal dysfunction [2]. We report a case of a 3-year-old girl with severe lead encephalo-neuropathy secondary to a pica activity and probably consumption of traditional medication, we discuss the clinical and public health issues surrounding lead toxicity in children.

## Patient and observation

**Patient information:** a 3-year-old female child was referred to the children's hospital of the University Hospital Center (CHU) in Casablanca in May 2018 for paraparesis. The child is of non-consanguineous parents, a housewife mother, and a traditional herbalist father. No family or personal pathological history. Indeed, her psychomotor development is normal according to her age, and correctly immunized.

**Clinical finding:** pyramidal syndrome with radicular involvement, bulbar and meningeal syndromes.

**Figure 1:**
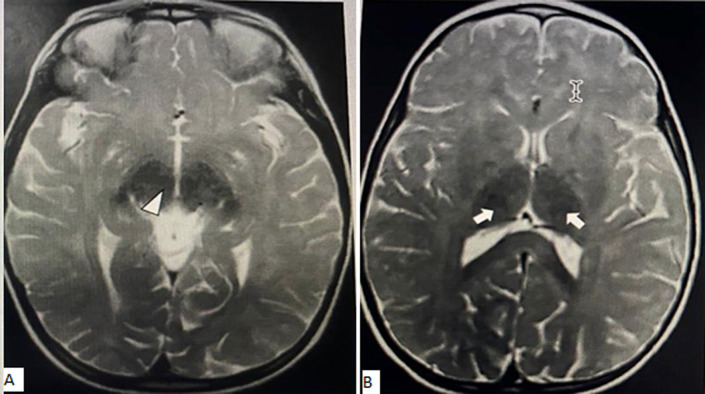
(A,B) brain magnetic resonance imaging thalamic (arrow) and diencephalic (arrow ahead) low signal T2

**Diagnostic assessment:** the clinical history date back to 12 days before admission by fever, arthralgia, myalgia, and dysphagia. this episode was treated as viral pharyngitis. On admission, the child was apyretic conscious but aphasic, slightly dehydrated, the heart rate (RH) was a beat slow at 55 beats/min, the respiratory rate (RR) at 32 C/min, and the oxygen saturation SaO_2_ (98%) were normal. The neurological examination found a child conscious but aphasic, sitting, and walking were impossible with a meningeal and axial stiffness. Tetra paresis with segmental muscle strength of 3/5. Spastic hypertonia of the upper limbs and weakness of the lower limbs. There were no sensory disturbances. Sharp osteotendinous reflexes in the upper limbs, normal in the lower limbs apart from bilateral abolition of the patellar reflex with bilateral Babinski's sign. Coordination was difficult to verify given the deficit. The child couldn't swallow her saliva; she indeed presented swallowing disorders with false routes; confirmed by the 3 sips test: the child was rejecting water. The remainder of the physical examination was normal. An initial biological assessment carried out included: a complete blood count (CBC) analysis that showed a normocytic normochromic anemia with High blood at 11.2 g/dl. The electrolyte assessment revealed a normal level of natremia, serum potassium, serum creatinine, blood urea and a normal C-reactive protein level. An encephalic magnetic resonance imaging (MRI) was carried out and objectified: a hypointense signal in T2 and flair sequence from the diencephalon to the bulb with dilation of the 4^th^ ventricle and a meningeal gadolinium intake at the level of the cervical cord and cortico-subcortical atrophy (Figure 1). Spinal, MRI did not show any abnormalities. Lumbar puncture collected clear cerebrospinal fluid with normal cytology; less than 3 elements/mm^3^, direct examination was negative, normal proteinorachia at 0.27g/l and glycorachia at 0.65 g/l. Bacteriological, virological and fungal study of cerebrospinal fluid for 14 pathogens was negative. The course was marked by the worsening of swallowing disorders and the appearance of partial seizures of the left upper limb. She was transferred to the pediatric intensive care unit; where the admission examination revealed a conscious child, T: 36.7 C, HR: 70 Beat/min, normal blood pressure (BP) at 90/60 mmHg, bradypnea at 12 C/min, SaO_2_ at 100% in the open air, with bronchial congestion, the child was still aphasic and quadriplegic. In front of the great atypia of the clinical picture associating at the same time central and radicular symptoms and the lack of any improvement under various administered treatments; the anamnesis was redone in the sense of a toxic origin; we found a notion of pica and probably an unacknowledged use of medications based on traditional medicine products. The broad toxicological screening was negative, but an elevated (BLL) at 176.4 μg/L was reported.

**Therapeutic intervention:** an oral lead chelator treatment was started by succimer (SUCCICAPTAL) at a dose of 100mg three times a day for 5 days, then 100mg twice a day for 15 days. The control (BLL) assay was not performed.

**Follow-up and outcomes:** sixteen (16) days after admission and 6 days after chelation treatment respiratory distress with respiratory pauses appeared. The child was restless, T: 37°C blood pressure (BP)= 90/60 mm Hg, heart rate (HR)= 170 beats/mn, thoraco-abdominal swinging with pauses in breathing requiring intubation and respiratory ventilation and later a tracheotomy. After a stay of more than 2 months in the intensive care unit, she finally unfortunately died from septic shock.

**Informed consent:** this work was performed in the university children´s hospital Ibn Rochd. University Hassan 2, Morocco with the collaboration of all the institutions of different authors cited above. The patient's father was orally informed, and his written consent was obtained.

## Discussion

Lead causes widespread effects on brain development and functional outcomes, it particularly affects the flow of calcium across cell membranes and the formation of microfilaments [3]. Tight junctions and other cell membrane structural components are damaged and ultimately break down, increasing the permeability of the blood-brain barrier and resulting in cerebral edema. As this process progresses, it manifests clinically as encephalopathy, seizures, coma, and ultimately death [3]. Lead encephalopathy is primarily due to lead´s effects on cerebrovascular endothelium, these effects occur at a high whole (BLL), generally greater than 80 to 100 microgram/dl [4]. In 2010, after reporting high mortality in young children exposed to environmental lead contamination in a village in northern Nigeria. Children ≤5 years were investigated for lead contamination with a threshold value of VBLLs ≥ 45 μg/dL as an inclusion criterion. There was evidence that a VBLL ≥80 μg/dL was associated with neurological features as seizures and/or altered consciousness, hyporeflexia, inconsolable crying, agitation, and decreased mobility. Neurological symptoms were also more likely in children aged 1-<3 years compared to those 3-5 years [5]. As opposed to nontoxic neuropathies, which are primarily sensory with involvement of the feet and distal regions first and then proximal regions later, whereas lead neuropathies are usually purely motor and the distribution of muscle weakness is characteristic with early and severe involvement of the wrist and finger extensors then other territories can be involved to a lesser degree [6]. In all cases the deficit is only or mainly motor: paresis and paralysis of the muscles of the arms, shoulders, thorax, lower limbs and the larynx (responsible for dysphonia) as in our patient [7]. In children, lead-related peripheral neuropathy is less common than in adults. In fact, our patient presented with paralysis of the lower limbs followed a week later by paralysis of the upper limbs. With rapidly progressive swallowing disorders with loss of walking within 48 hours. In a review of the English literature of reported cases of lead-related neuropathy in children; several cases of children aged between two and half (2.5) and 7 years, of both sexes, presented with heaviness in both inferior and superior limbs with difficulty or even loss of walking even going as far as flaccid quadriplegia with breathing difficulties have been described. The duration of onset and progression of symptoms ranged from 2 to 3 weeks and up to a few months. Our patient presented the same clinical picture as those reported in this study [8]. The symptomatology of lead-related neuropathy is suggestive of Guillain-Barré syndrome if the practitioners are not aware of this pathology. Measurement of porphyrin or lead concentrations should be done to diagnose unusual causes of acute neuropathy not caused by Guillain-Barre syndrome [9].

## Conclusion

Lead poisoning must be taken into account in all unexplained cases especially in neuropathy mimicking Guillain-Barre syndrome mainly among individuals of lower socioeconomic classes and in children with specific psychopathological behaviors. Given that there is no safe level of lead poisoning for children and that chelating agents have a limited value in reducing the effects of lead poisoning, primary prevention is therefore paramount.
